# Immunobiology and Pharmacologic Manipulation of Dendritic and Regulatory Cells

**DOI:** 10.1155/2013/186983

**Published:** 2013-06-26

**Authors:** Mohamad Mohty, Arnon Nagler, Nicolaus Kröger, Beatrice Gaugler

**Affiliations:** ^1^Université Pierre et Marie Curie (UPMC), 75012 Paris, France; ^2^Hematology Department, Hôpital Saint-Antoine, APHP, 75012 Paris, France; ^3^INSERM UMR_S 938, 75012 Paris, France; ^4^Tel Aviv University, Chaim Sheba Medical Center, 52621 Tel Hashomer, Israel; ^5^Department of Stem Cell Transplantation, University Hospital Hamburg-Eppendorf, 20246 Hamburg, Germany; ^6^INSERM UMR1098, EFS Bourgogne Franche-Comté, Université de Franche-Comté, 25000 Besançon, France

Antigen presenting cells (APCs), especially dendritic cells (DCs), play a major role in the hierarchy of the induction of immune reactions. DCs are widely accepted as the most potent APCs capable of inducing protective adaptive immune responses in addition to tolerance to self-antigens. The role of DCs is currently being investigated in the context of many disease and therapeutic settings. In response to a variety of microbial and endogenous stimuli, resting DCs in peripheral tissues undergo a complex maturation process that might involve the regulation of genes that control distinct DC functions. The different functional properties of DCs are also linked to the existence of several subpopulations in humans and animals that differ in response to stimuli. In this special issue, several articles addressed some recent findings highlighting the interactions between key immune effectors both in vitro and in vivo and in different disease settings. 

V. R. Yanofsky et al. provided an in-depth analysis of DC biology, with a particular focus on skin DCs and their role in cutaneous carcinoma. C. Y. J. Chung et al. provided an overview of the role of DCs in the immunopathogenesis of autoimmunity, as well as recent concepts of DC-based therapeutic opportunities in autoimmune diseases. On the other hand, P. Rúiz et al. described the different treatments and some of the novel immunotherapeutic strategies undertaken to induce transplantation tolerance in general. More specifically, M. Michael et al. discussed the current knowledge of Treg biology and its potential for cell-based immunotherapy in allogeneic stem cell transplantation. The review by A. K. Gloudemans et al. summarized the latest literature on the role of mucosal IgA in protection against allergic airway disease, the mechanisms described to induce secretory IgA, and the role of (mucosal) DCs in this process.

D.-Y. Chen et al. examined the effect of dextromethorphan (DXM), a common cough suppressant, on the activation and function of DCs. Interestingly, DXM decreased the LPS-induced surface expression of CD80, CD83, and HLA-DR and the secretion of IL-6 and IL-12 in human monocyte-derived DCs. These findings provide a new insight into the impact of DXM treatment on DCs and suggest that DXM has the potential to be used in treating DC-related acute and chronic diseases. Similarly, L. Adalid-Peralta et al. showed that cysticerci may modulate DCs to favor a suppressive environment, which may help parasite establishment, minimizing the excessive inflammation, which may lead to tissue damage. In the same line, R. N. Ramos et al. described that cytokines such as IL-10 and TGF-beta, as well as cell surface molecules like PD-L1 and ICOS, seem to be significantly involved in the redirection of DCs towards tolerance induction, and tumor cells may modulate distinct DC subpopulations through the involvement of these molecules. Another work from X. Gu et al. suggested that regulation of B7-H1 expression on hepatic stellate cells by IFN-*γ* represents an important mechanism that regulates immune responses in the liver favoring tolerogenicity rather than immunogenicity. Finally, A. Brosbøl-Ravnborg et al. described how vitamin D3 and TLR agonists acted in synergy to alter secretion of cytokines from human DCs in a direction that may provide an anti-inflammatory environment. 

From a therapeutic perspective, C. Penna et al. elegantly showed that a combined treatment, including dexamethasone preconditioning followed by an inoculation of short-term LPS-stimulated type II collagen-loaded DCs, provides an improved strategy for attenuating arthritis severity. On the other hand, data from S. O. Åkefeldt et al. suggested that targeting MCL1 and BCL2A1 in infiltrating DC may affect the clinical outcomes in rheumatoid arthritis (RA) and Langerhans cell histiocytosis (LCH). P. Cordiali-Fei et al. showed that in patients with limited systemic sclerosis, Treg cells were inversely correlated to disease duration, suggesting that their levels may represent a marker of disease progression. Interestingly, C. Doñas et al. showed that Trichostatin A (TSA) could potentially be used to enhance the differentiation and suppressive function of CD4+Foxp3+ Treg cells.

Finally, L. de la Cruz-Merino et al. reviewed some important aspects about the role of tumor-infiltrating lymphocytes (TIL) and their subtypes, tumor-associated macrophages (TAM), and myeloid-derived suppressive cells (MDSC) that will eventually be incorporated into diagnostic and therapeutic algorithms of breast cancer. The work from W. Maes et al. discussed based on an animal model the value of local elimination of Tregs within the tumor microenvironment which might represent an important tool from both fundamental and clinical perspectives.

All in all, articles published in this special issue clearly show that DCs and Tregs are currently being considered as attractive targets towards manipulation of the immune system for therapeutic purposes in different human disease settings.

*Mohamad Mohty* *Arnon Nagler* *Nicolaus Kröger* *Beatrice Gaugler*

